# Sex differences in coronary microvascular resistance measured by a computational fluid dynamics model

**DOI:** 10.3389/fcvm.2023.1159160

**Published:** 2023-07-06

**Authors:** Daniel J. Taylor, Louise Aubiniere-Robb, Rebecca Gosling, Tom Newman, D. Rodney Hose, Ian Halliday, Patricia V. Lawford, Andrew J. Narracott, Julian P. Gunn, Paul D. Morris

**Affiliations:** ^1^ Department of Infection, Immunity and Cardiovascular Disease, University of Sheffield, Sheffield, United Kingdom; ^2^ Department of Cardiology, Sheffield Teaching Hospitals NHS Foundation Trust, Sheffield, United Kingdom; ^3^ Insigneo Institute for in Silico Medicine, University of Sheffield, Sheffield, United Kingdom

**Keywords:** coronary microvascular resistance, sex, computational fluid dynamics, coronary microvascular dysfunction, coronary physiology

## Abstract

**Background:**

Increased coronary microvascular resistance (CMVR) is associated with coronary microvascular dysfunction (CMD). Although CMD is more common in women, sex-specific differences in CMVR have not been demonstrated previously.

**Aim:**

To compare CMVR between men and women being investigated for chest pain.

**Methods and results:**

We used a computational fluid dynamics (CFD) model of human coronary physiology to calculate absolute CMVR based on invasive coronary angiographic images and pressures in 203 coronary arteries from 144 individual patients. CMVR was significantly higher in women than men (860 [650–1,205] vs. 680 [520–865] WU, *Z* = −2.24, *p* = 0.025). None of the other major subgroup comparisons yielded any differences in CMVR.

**Conclusion:**

CMVR was significantly higher in women compared with men. These sex-specific differences may help to explain the increased prevalence of CMD in women.

## 1. Introduction

In health, the epicardial coronary arteries act as low resistance conductance vessels, whereas the distal microvessels exhibit dynamic resistance, variation in which matches coronary blood flow (CBF) closely to the prevailing metabolic demands of the myocardium. Pathological increases in the resistance of either compartment can reduce maximal CBF, resulting in ischaemia. Unlike epicardial disease, the investigation and treatment of coronary microvascular dysfunction (CMD) is less well established. In many cases, CMD is associated with increased coronary microvascular resistance (CMVR) (1). CMD is common in patients with epicardial coronary artery disease (CAD) and in those with angina with no obstructive epicardial disease (ANOCA) (2), with a recent meta-analysis suggesting a prevalence of 41% in the latter group (3). Furthermore, In the CE-MARC2 coronary physiology sub-study, Corcoran et al. found that, in patients undergoing invasive assessment for suspected CAD, 68% had some evidence of impaired coronary microvascular physiology, with similarly high rates in those with obstructive CAD (4). When CMD reduces the maximum vasodilatory reserve of the coronary circulation, which may be measured using coronary flow reserve (CFR), it is associated with an increased likelihood of major adverse cardiac events (5). Similarly, microvascular assessment has prognostic value in the assessment of patients with both acute coronary syndrome (ACS) (6) and chronic coronary syndrome (CCS) (7). CMD can be treated with guideline-indicated therapy, with improvements in angina, quality of life and illness perception (8). Studies show CMD is more common in women than men (9–11). However, there are no data showing sex differences in CMVR. The aim of this study was to compare hyperaemic CMVR in men vs. women and investigate other major subgroups in patients undergoing angiography for the investigation of chest pain.

## 2. Methods

### 2.1. Patient recruitment

Patients undergoing cardiac catheterisation for acute and chronic coronary syndromes at Sheffield Teaching Hospitals NHS Foundation Trust were considered eligible. For acute cases, only non-culprit arteries were considered. Further exclusion criteria for all cases included ST-segment elevation myocardial infarction within the preceding 60 days, any contraindication to adenosine or contrast media, previous coronary artery bypass surgery, patient age below 18 years, chronic total occlusion, severe valvular disease and an inability to consent. This was a *post hoc* analysis of the Complementary Value of Absolute Coronary Flow in the Assessment of Patients with Ischaemic Heart Disease (the COMPAC-Flow) study (12), in which computational fluid dynamics- (CFD-) derived absolute flow reduction in CAD was assessed using the virtuQ™ software package (13). The study was approved by Regional Ethics Committees (16/NW/0897 and 08/H1308/193) and informed consent was obtained.

### 2.2. Clinical data collection

Coronary angiography and FFR assessment was performed using standard techniques. During angiography, operators were encouraged to acquire clear images of the vessel of interest, with minimal overlap, panning and foreshortening, to optimise computational arterial reconstruction (14). Translesional pressure measurements under hyperaemic and baseline conditions were taken with either the PressureWire X (Abbott Laboratories) or PrimeWire Prestige (Philips Volcano). Hyperaemia was achieved with an intravenous infusion of adenosine 140 µg/kg/min. Pseudoanonymised angiography (DICOM), physiological (pressure) and other clinical data were exported to the University of Sheffield for computational processing and analysis.

### 2.3. Simulating coronary flow and CMVR

A full description of the virtuQ workflow, including arterial reconstruction, has previously been published (13, 15). In summary, two angiographic projections taken at least 30° apart were used to produce a 3D, axisymmetric, rigid reconstruction of the coronary artery of interest from an epipolar line method. Arteries with no appreciable stenosis were excluded, because the CFD method requires an epicardial pressure gradient to derive the flow and resistance values (13, 16). The quality of arterial reconstructions was assessed by three cardiologists who were also expert users of the virtuQ software, all of whom were blinded to the CFD results. Invasive pressure measurements, corresponding to proximal (Pa) and distal (Pd) measurements were prescribed at the reconstruction inlet and outlet respectively to define boundary conditions. A CFD simulation was then performed, resolving the Navier-Stokes and continuity equations to yield absolute coronary blood flow (*Q*_CFD_), in ml/min, at the outlet of the reconstructed artery under both hyperaemic and baseline conditions. CFD simulations used standard blood parameters (density 1,056 kg/m^3^; viscosity 0.0035 Pa s) and modelled steady, laminar flow of a Newtonian fluid, the suitability of which has previously been demonstrated (17–19). Computed CMVR (CMVR_CFD_) was calculated using the hydraulic equivalent of Ohm's law:

$$\mathrm{CMV}R_{\mathrm{CFD}}=1,000\frac{\mathrm{Pd}}{Q_{\mathrm{CFD}}}.$$

**Figure 1:**
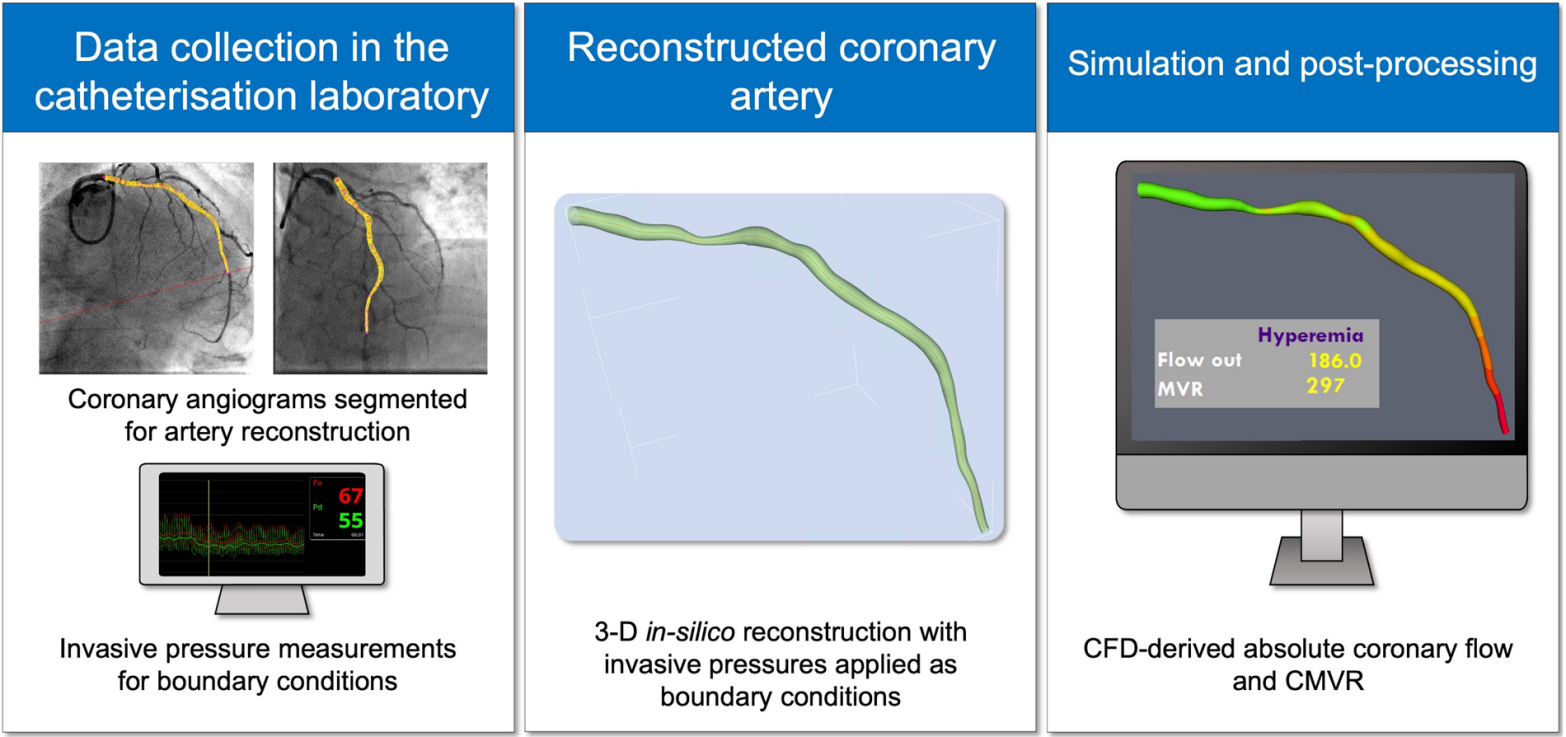
Schematic of CFD workflow.

A conversion factor of 1,000 was applied to yield CMVR_CFD_ results in Woods units (WU) (Figure 1). Computed CFR (CFR_CFD_) was calculated as the ratio of hyperaemic and baseline *Q*_CFD_:

$$\mathrm{CF}R_{\mathrm{CFD}}=\frac{\mathrm{Hyperaemic}\;Q_{\mathrm{CFD}}}{\mathrm{Baseline}\;Q_{\mathrm{CFD}}}.$$

### 2.4. Statistical analysis

Categorical variables are presented as frequency (percentage). The Shapiro–Wilk test was used to assess the spread of data. Normally distributed continuous variables are presented as mean ± standard deviation, while skewed data are presented as median [interquartile range]. Continuous values of haemodynamic parameters were compared using the unpaired *t*-test, Mann–Whitney *U*, one-way ANOVA and Kruskal–Wallis tests where appropriate, categorical variables were compared with Chi Square. Cohen's *d* and Hedges' *g* were used to compare effect size between two samples as indicated. Correlation was quantified using Pearson's correlation coefficient (*r*). A statistical threshold of *p* = 0.05 was considered significant and all statistical tests were two-tailed. The primary endpoint was a comparison of the CMVR_CFD_ between men and women. The secondary endpoints were comparisons of other major subgroups.

## 3. Results

### 3.1. Patient characteristics

From a potential 169 patients, 144 were included. Of these, 109 were male (76%), mean age was 65 ± 10 years and 129 patients were white Caucasian. Ninety-two (64%) patients were overweight (BMI > 25) and 91 (63%) had a history of smoking. Further details of demographics and comorbidities shown in Table 1.

**Table 1:** Recruited patient characteristics.

Demographics	
Number of patients	144
Age (years)	65 ± 10
Male gender	109 (76%)
White Caucasian	129 (90%)
Current or previous smoker	91 (63%)
BMI >25	92 (64%)
Comorbidities	
Hypertension	94 (65%)
Dyslipidaemia	109 (76%)
Diabetes mellitus	37 (26%)
Chronic lung disease	16 (11%)
Valvular heart disease	7 (5%)
Previous myocardial infarction	36 (25%)
Left ventricular systolic dysfunction	29 (20%)
Arteries	
LAD	103 (51%)
RCA	45 (22%)
LCx	26 (13%)
Dx	17 (8%)
OM	7 (3%)
LMS	5 (2%)

Data presented as absolute number (%) or mean ± standard deviation. BMI, body mass index; Dx diagonal; LAD, left anterior descending; LCx, left circumflex; LMS, left main stem; OM, obtuse marginal; RCA, right coronary artery.

### 3.2. Artery characteristics and case exclusions

**Figure 2:**
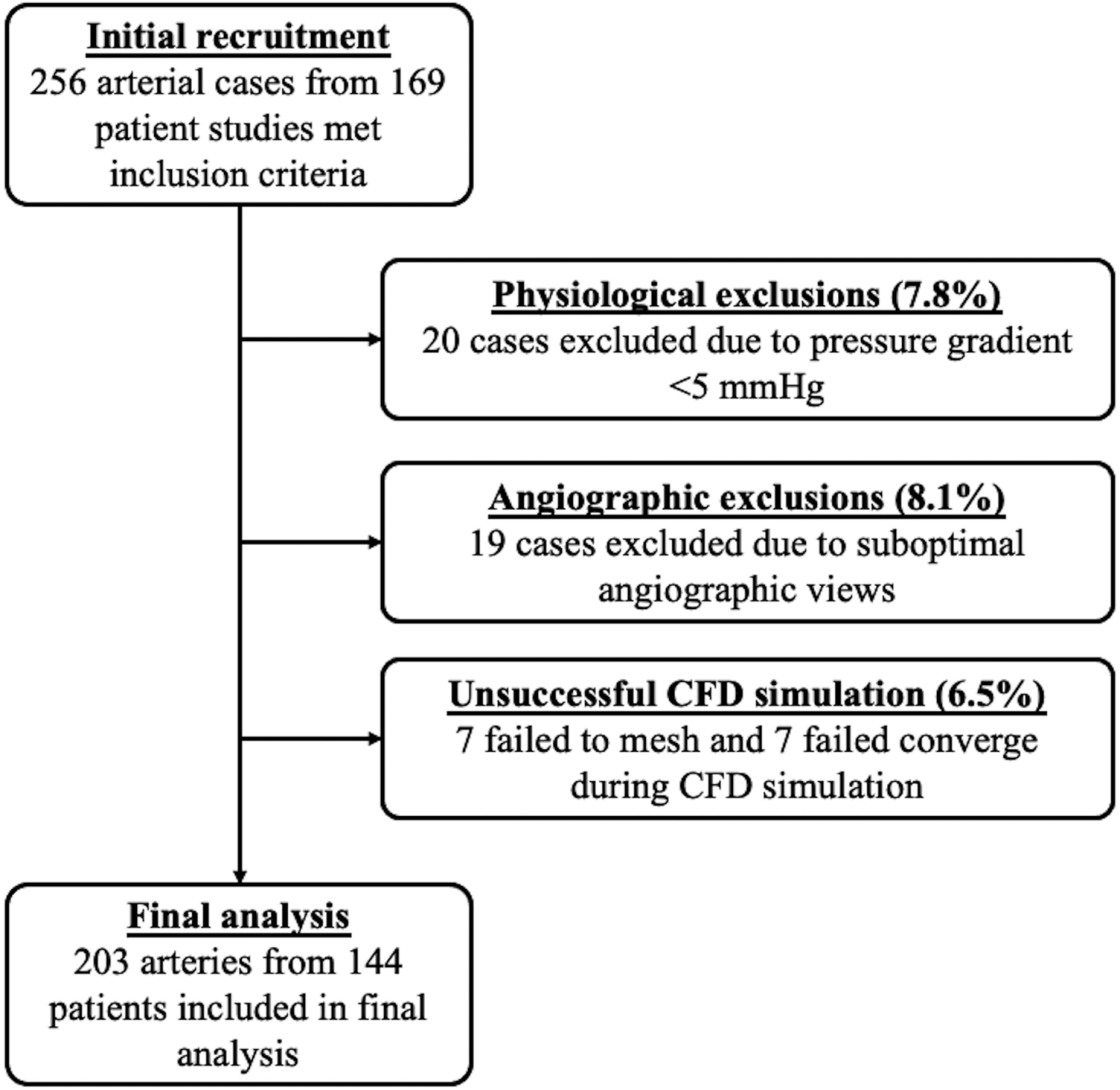
Consort diagram.

From a potential 256 arterial cases, 203 were included. These comprised 103 left anterior descending (LAD) arteries, 45 right coronary arteries (RCA), 26 left circumflex (LCx) arteries, 17 diagonal (Dx) arteries, seven obtuse marginal (OM) arteries and five left main stem (LMS) arteries. Median CMVR_CFD_ for all cases was 710 [515–980] WU. The median FFR was 0.80 [0.72–0.87] and median visually assessed lesion stenosis was 60 [50%–70%]. Cases were excluded due to inadequate pressure gradients for CFD simulation (*n* = 20), inadequate angiographic views for arterial reconstruction (*n* = 19), failure of volumetric meshing (pre-requisite for CFD simulation, *n* = 7), failure of CFD simulation convergence (*n* = 7). All 203 included cases yielded CMVR_CFD_ results. See Figure 2 for a full consort diagram.

### 3.3. Comparison of CMVR_CFD_ between key patient groups

**Figure 3:**
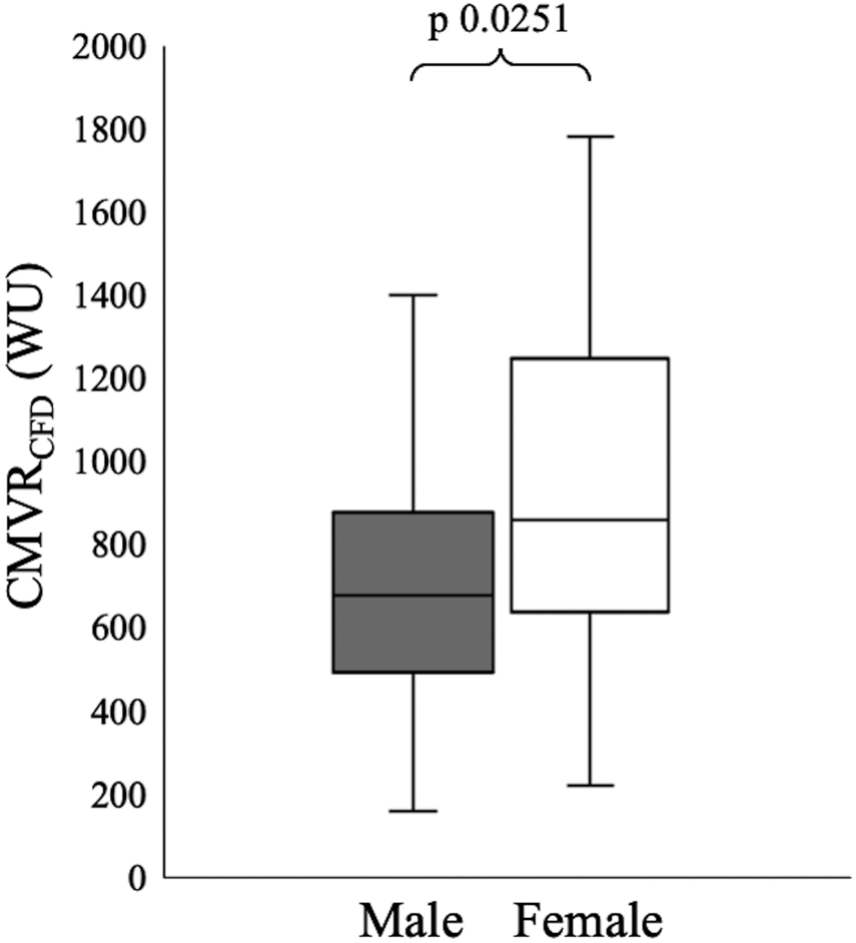
CMVRCFD was significantly higher in females versus males. WU, Woods units.

Hyperaemic CMVR_CFD_ was significantly higher in women 860 [650–1,205] WU vs. men 680 [520–865] WU (*Z* = −2.24, *p* = 0.02). The effect of this difference was small (Hedges' *g* = 0.35) (Figure 3). There were no significant differences between male and female patients for any demographic or comorbidity variables, with comparable FFR (women 0.80 [0.72–0.87], men 0.81 [0.72–0.89], *Z* = −0.85, *p* = 0.40) and percentage lesion stenosis (women 60% [50%–70%], men 60% [50%–70%], *Z* = 0.05, *p* = 0.96). Further analysis using baseline conditions, revealed resting CMVR_CFD_ was also significantly higher in women 1,765 [1,260–2,713] WU vs. men 1,370 [990–2,020] (*Z* = −2.46, *p* = 0.01, Hedges’ g = 0.46), but CFR_CFD_ did not vary between the sexes (women 1.74 [1.35–2.30] vs. men 1.61 [1.32–1.98], *Z* = −0.48, *p* = 0.63) (Table 2).

**Table 2:** Differences in male and female characteristics.

Demographics	Male	Female	*p*
Number of patients	109	35	
Age (years)	64 ± 10	67 ± 10	0.072
White Caucasian	97 (89%)	32 (91%)	0.43
Current or previous smoker	69 (63%)	23 (66%)	0.80
BMI >25	61 (56%)	18 (51%)	0.85
Comorbidities			
Hypertension	71 (65%)	23 (66%)	0.95
Dyslipidaemia	81 (74%)	28 (80%)	0.49
Diabetes mellitus	27 (25%)	10 (29%)	0.59
Chronic lung disease	12 (11%)	4 (11%)	0.95
Valvular heart disease	5 (5%)	2 (6%)	0.79
Previous myocardial infarction	29 (27%)	7 (20%)	0.43
LVSD	24 (37%)	5 (31%)	0.67
Lesion characteristics			
FFR	0.80 [0.72–0.87]	0.81 [0.72–0.89]	0.40
Percentage stenosis	60 [50–70]	60 [50–70]	0.96
Hyperaemic CMVR_CFD_ (WU)	680 [520–865]	860 [650–1,205]	0.025
Baseline CMVR_CFD_ (WU)	1,370 [990–2,020]	1,765 [1,260–2,713]	0.014
Hyperaemic *Q*_CFD_ (ml/min)	87 [65–120]	77 [63–106]	0.28
Baseline *Q*_CFD_ (ml/min)	52 [39–74]	48 [33–62]	0.23
CFR_CFD_	1.60 [1.32–1.97]	1.74 [1.35–2.30]	0.63

Data presented as absolute number (%), mean ± standard deviation or median [IQR]. LVSD, left ventricular systolic dysfunction.

CMVR_CFD_ was not influenced by smoking status (*Z* = −0.93, *p* = 0.35), body mass index (BMI) > 25 (*Z* = −1.06, *p* = 0.30), hypertension (*Z* = 0.54, *p* = 0.59), dyslipidaemia (*Z* = −0.48, *p* = 0.63), diabetes (*Z* = −0.72, *p* = 0.47), chronic lung disease (*Z* = −0.11, *p* = 0.92), valvular heart disease (*Z* = −1.12, *p* = 0.26), previous myocardial infarction (*Z* = 1.39, *p* = 0.16) or left ventricular systolic dysfunction (*Z* = 1.55, *p* = 0.12) (Table 3). No significant correlations were identified between CMVR_CFD_ with age (*r* =* *−0.08, *p* = 0.25), estimated glomerular filtration rate (*r* =* *0.12, *p* = 0.10), haemoglobin concentration (*r* =* *−0.07, *p* = 0.32) or haematocrit (*r* =* *−0.06, *p* = 0.45). CMVR_CFD_ was also higher in patients of black and Asian ethnicity 985 [810–1,303] WU vs. white Caucasian patients 690 [520–890] WU (*Z* = −2.18, *p* = 0.03) (Supplementary Material). However, the number of patients within the black and Asian group was only eight.

**Table 3:** Comparison of hyperaemic CMVR_CFD_ between key subgroups.

Variable	CMVR_CFD_			Statistic	*p*
Sex	Male 680 [520–865]		Female 860 [650–1,205]	*z* = −2.24	0.0251*
Ethnicity	White Caucasian 690 [520–890]		Black/Asian 985 [810–1,303]	*z* = −2.18	0.0293*
Current/previous smoker	No 778 [563–1,123]		Yes 700 [546–933]	*z* = −0.93	0.352
BMI >25	No 763 [558–1,253]		Yes 665 [450–885]	*z* = −1.06	0.289
Hypertension	No 685 [565–870]		Yes 720 [529–1,013]	*z* = 0.54	0.589
Dyslipidaemia	No 720 [605–885]		Yes 700 [490–980]	*z* = −0.48	0.631
Diabetes mellitus	No 715 [525–920]		Yes 680 [583–1,020]	*z* = −0.72	0.472
Chronic lung disease	No 713 [551–950]		Yes 745 [533–983]	*z* = −0.11	0.920
Valvular heart disease	No 700 [520–970]		Yes 800 [708–1,000]	*z* = −1.12	0.263
Previous MI	No 720 [559–1,071]		Yes 650 [485–831]	*z* = 1.39	0.165
LVSD	No 770 [595–1,133]		Yes 670 [430–805]	*z* = 1.55	0.121
Coronary artery origin	Left 720 [543–1,018]		Right 640 [440–930]	*z* = 1.78	0.0724
Coronary artery	LAD 720 [550–988]	LCx 780 [570–1,170]	RCA 640 [440–930]	*H* = 4.19	0.123
					

BMI, body mass index; LAD, left anterior descending; LCx, left circumflex; LVSD, left ventricular systolic dysfunction; MI, myocardial infarction; RCA, right coronary artery.

*Denotes statistically significant result.

### 3.4. Inter-artery comparison of CMVR_CFD_

CMVR_CFD_ did not differ between arteries originating from, and including, the LMS 720 [543–1,018] WU vs. the RCA 640 [440–930] WU (*Z* = 1.80, *p* = 0.07). Inter-artery comparison did not show a significant difference in CMVR_CFD_ between the LAD and main diagonal branch 720 [550–988] WU vs. the LCx and obtuse marginal branch 780 [570–1,170] WU vs. RCA 640 [440–930] WU (*H* = 4.19, *p* = 0.12) (Table 2).

## 4. Discussion

In this study, we analysed absolute CMVR_CFD_ derived from invasive pressure measurements using CFD simulation in 203 coronary arteries from 144 patients (109 male, 35 female). CMR_CFD_ was significantly higher in women when compared to men. There were no other significant differences comparing major sub-groups. CMVR_CFD_ was higher in those of black and Asian vs. Caucasian ethnicity, but this group was very small (*n* = 8).

### 4.1. Subgroup differences in CMVR

Despite the well-established increased prevalence of CMD in women (9–11) and the numerous techniques for quantifying CMVR (13, 20–23), no previous study has demonstrated a significant sex-specific difference in CMVR. Prior studies have shown no difference in the index of microvascular resistance (IMR) between men and women (24, 25), with apparent discrepancies in microvascular function attributed to lower CFR in women as a result of elevated baseline coronary flow (26). Our study, therefore, provides the first observation of a sex-specific difference in CMVR, suggesting a true microvascular dysfunction may contribute to sex-specific differences in CMD. The reasons underpinning these discrepancies are currently unknown, with a lack of absolute flow results (ml/min) in previous studies hindering comparisons. Differences in enrollment may have contributed; prior studies predominantly included patients with ANOCA (mean lesion percentage stenosis ranged from 20% to 30%), while our study included a large proportion of patients with haemodynamically significant CAD. The hyperaemic flow values quoted in this study are lower than previously measured with the Rayflow catheter in patients with ANOCA (27) and it is possible the flow limiting effect of epicardial stenoses blunted any sex-specific differences in CFR. Furthermore, prior studies used the mean transit time (MTT) of an intracoronary saline bolus as a surrogate for coronary flow (IMR =* *distal pressure × MTT) (24, 25), a technique which is subject to significant error (28). The direction of this effect appears cogent with the fact that women have a higher prevalence of CMD and that CMVR and CMD are associated (9–11). The underlying mechanism(s) behind sex differences in microvascular function are largely unknown. Some data suggest changes in sex hormones, particularly in the peri- and post-menopausal periods may contribute to coronary endothelial dysfunction and abnormal vasomotor control (29) and this does appear to be consistent with clinical practice. In our study, the mean age of female participants was 67 ± 10 years old (the men were 64 ± 10 years old), making menopause-induced microvascular changes a plausible explanation for the observed difference between sexes. In the females, age was not correlated with CMVR_CFD_ (*r* =* *−0.22, *p* = 0.20); but as only four patients were less than 55 years old, we could not determine whether CMVR differed between the peri- and post-menopausal groups. We also demonstrated a statistically significant difference in CMVR_CFD_ between white Caucasian vs. black and Asian patients. This however, was based upon only eight patients and so these results are unreliable.

### 4.2. Clinical implications

Despite being described over thirty years ago (30), CMD continues to pose a clinical challenge. Angiography alone is good at excluding epicardial disease, but is unable to diagnose CMD. Both European and American guidelines now recommend invasive assessment of CFR or IMR to support diagnosis (31, 32). CFR alone does not discriminate between epicardial and microvascular compartments, whereas indices of microvascular resistance require combined pressure and flow measurements. While the measurement of intracoronary pressure is simple, accurate and reproducible, estimating coronary flow is more challenging. Traditionally, a surrogate of flow rate was inferred from either Doppler flow velocity or the MTT of in injected bolus of room temperature saline (thermodilution). Both these techniques for estimating coronary flow are subject to variability; Doppler readings are dependent upon sensor alignment with the direction of flow and proximity to the vessel wall (33), whilst bolus thermodilution is dependent upon injection quality and is unsuitable for some bradycardic patients and is affected by side branch flow (34). Recent work has demonstrated poor agreement between Doppler and thermodilution derived CFR (mean bias 0.59 ± 1.24; *R*^2 ^= 0.36, *p* < 0.0001) and microvascular resistance (*R*^2 ^= 0.19; *p* < 0.0001) even in expert hands (28). The continuous infusion thermodilution technique, using a the Rayflow™ catheter, provides an alternative method of invasively deriving absolute coronary blood flow and microvascular resistance that delivers better reproducibility (35). All invasive measurements add time and expense to a standard angiogram and this may affect widespread uptake. The current results are not entirely consistent with previous work suggesting CMD in women is a functional phenotype characterized by a decreased CFR with increased resting flow but maintained hyperaemic flow and resistance (24, 25). Given the observational nature of our study and the potential limitations of the methodology, further work is needed to corroborate the findings and evaluate prognostic significance. The method for quantifying CMVR_CFD_ in this study does however, allow for real-time assessment of the coronary microcirculation from a simple angiography and a standard FFR assessment and may influence future approaches in coronary physiological assessments.

### 4.3. Study limitations

First, more men were recruited than women. However, this is not unusual in studies of CAD. Second, patients with completely normal epicardial arteries were excluded. This is likely to have reduced the numbers of patients with ANOCA. This is important because it may have reduced the magnitude of the observed differences. Future studies of sex-specific differences in CMVR should also include ANOCA patients and not exclude patients with unobstructed epicardial arteries. The computational method used in this study did not account for side-branch flow, subtended myocardial mass or collateral blood supply (36). This may also have influenced CMVR_CFD_ results, but is unlikely to have influenced between-group differences. Although the CFD method has been validated *in vitro*, the physiological calculations may be subject to several sources of inaccuracies introduced from both invasive pressure measurements and the various stages of the CFD workflow, not least the *in silico* arterial reconstruction (15, 16). Model sensitivity to these various sources of error is yet to be fully quantified and is likely to be case specific. For example, in minimally-stenosed arteries, geometric error in the reference vessel is a dominant source of inaccuracy (16). In stenosed arteries, any error in the 3D reconstruction around the region of the stenosis will contribute significantly to overall model error (37). Gravitational error of invasively measured pressure was not corrected for, which will also contribute error (38, 39), albeit to a lesser extent in increasingly stenosed cases.

## 5. Conclusion

In this single center study, using a computational method, we have demonstrated sex-specific differences in calculated CMVR, in patients under invasive investigation for chest pain. These findings suggest hyperaemic CMVR may be higher in women than men and may help to explain the higher prevalence of CMD in women. Further investigation and studies are required to confirm these findings.

## Data Availability

The original contributions presented in the study are included in the article/**Supplementary Material**, further inquiries can be directed to the corresponding author.

## References

[1] Morris PD Al-Lamee RK Berry C. Coronary physiological assessment in the catheter laboratory: haemodynamics, clinical assessment and future perspectives. Heart. (2022) 108(21):1737–46. 10.1136/heartjnl-2020-318743 35768192 PMC9606498

[2] Patel MR Peterson ED Dai D Brennan JM Redberg RF Anderson HV Low diagnostic yield of elective coronary angiography. N Engl J Med. (2010) 362(10):886–95. 10.1056/NEJMoa0907272 20220183 PMC3920593

[3] Mileva N Nagumo S Mizukami T Sonck J Berry C Gallinoro E Prevalence of coronary microvascular disease and coronary vasospasm in patients with nonobstructive coronary artery disease: systematic review and meta-analysis. J Am Heart Assoc. (2022) 11(7):e023207. 10.1161/JAHA.121.023207 35301851 PMC9075440

[4] Corcoran D Young R Adlam D McConnachie A Mangion K Ripley D Coronary microvascular dysfunction in patients with stable coronary artery disease: the CE-MARC 2 coronary physiology sub-study. Int J Cardiol. (2018) 266:7–14. 10.1016/j.ijcard.2018.04.061 29716756 PMC6008494

[5] Lee JM Jung J-H Hwang D Park J Fan Y Na S-H Coronary flow reserve and microcirculatory resistance in patients with intermediate coronary stenosis. J Am Coll Cardiol. (2016) 67(10):1158–69. 10.1016/j.jacc.2015.12.053 26965536

[6] Jin X Yoon MH Seo KW Tahk SJ Lim HS Yang HM Usefulness of hyperemic microvascular resistance index as a predictor of clinical outcomes in patients with ST-segment elevation myocardial infarction. Korean Circ J. (2015) 45(3):194–201. 10.4070/kcj.2015.45.3.194 26023307 PMC4446813

[7] Nishi T Murai T Ciccarelli G Shah SV Kobayashi Y Derimay F Prognostic value of coronary microvascular function measured immediately after percutaneous coronary intervention in stable coronary artery disease: an international multicenter study. Circ Cardiovasc Interv. (2019) 12(9):e007889. 10.1161/CIRCINTERVENTIONS.119.007889 31525096

[8] Ford TJ Stanley B Good R Rocchiccioli P McEntegart M Watkins S Stratified medical therapy using invasive coronary function testing in angina: the CorMicA trial. J Am Coll Cardiol. (2018) 72(23 Pt A):2841–55. 10.1016/j.jacc.2018.09.006 30266608

[9] Daly C Clemens F Lopez Sendon JL Tavazzi L Boersma E Danchin N Gender differences in the management and clinical outcome of stable angina. Circulation. (2006) 113(4):490–8. 10.1161/CIRCULATIONAHA.105.561647 16449728

[10] Bugiardini R Bairey Merz CN. Angina with “normal” coronary ArteriesA changing philosophy. JAMA. (2005) 293(4):477–84. 10.1001/jama.293.4.477 15671433

[11] Humphries KH Pu A Gao M Carere RG Pilote L. Angina with “normal” coronary arteries: sex differences in outcomes. Am Heart J. (2008) 155(2):375–81. 10.1016/j.ahj.2007.10.019 18215611

[12] Aubiniere-Robb L Gosling R Taylor DJ Newman T Rodney D Ian Halliday H The complementary value of absolute coronary flow in the assessment of patients with ischaemic heart disease (the COMPAC-flow study). Nat Cardiovasc Res. (2022) 1(7):611–6. 10.1038/s44161-022-00091-z 35865080 PMC7613105

[13] Morris PD Gosling R Zwierzak I Evans H Aubiniere-Robb L Czechowicz K A novel method for measuring absolute coronary blood flow & microvascular resistance in patients with ischaemic heart disease. Cardiovasc Res. (2020) 117(6):1567–77. 10.1093/cvr/cvaa220 PMC8152717 32666101

[14] Ghobrial M Haley HA Gosling R Rammohan V Lawford PV Hose DR The new role of diagnostic angiography in coronary physiological assessment. Heart. (2021) 107(10):783. 10.1136/heartjnl-2020-318289 33419878 PMC8077221

[15] Solanki R Gosling R Rammohan V Pederzani G Garg P Heppenstall J The importance of three dimensional coronary artery reconstruction accuracy when computing virtual fractional flow reserve from invasive angiography. Sci Rep. (2021) 11(1):19694. 10.1038/s41598-021-99065-7 34608218 PMC8490364

[16] Taylor DJ Feher J Czechowicz K Halliday I Hose DR Gosling R Validation of a novel numerical model to predict regionalized blood flow in the coronary arteries. Eur Heart J Digit Health. (2023) 4(2):81–9. 10.1093/ehjdh/ztac077 36974271 PMC10039427

[17] Morris PD. Computational fluid dynamics modelling of coronary artery disease. Sheffield: University of Sheffield (2015). https://etheses.whiterose.ac.uk/11772/

[18] Huckaba CE Hahn AW. A generalized approach to the modeling of arterial blood flow. Bull Math Biophys. (1968) 30(4):645–62. 10.1007/BF02476681 5701217

[19] Brown AG Shi Y Marzo A Staicu C Valverde I Beerbaum P Accuracy vs. computational time: translating aortic simulations to the clinic. J Biomech. (2012) 45(3):516–23. 10.1016/j.jbiomech.2011.11.041 22189248

[20] Fearon WF Balsam LB Farouque HM Caffarelli AD Robbins RC Fitzgerald PJ Novel index for invasively assessing the coronary microcirculation. Circulation. (2003) 107(25):3129–32. 10.1161/01.CIR.0000080700.98607.D1 12821539

[21] Nolte F van de Hoef TP Meuwissen M Voskuil M Chamuleau SA Henriques JP Increased hyperaemic coronary microvascular resistance adds to the presence of myocardial ischaemia. EuroIntervention. (2014) 9(12):1423–31. 10.4244/EIJV9I12A240 24755383

[22] De Bruyne B Pijls NHJ Gallinoro E Candreva A Fournier S Keulards DCJ Microvascular resistance reserve for assessment of coronary microvascular function: JACC technology corner. J Am Coll Cardiol. (2021) 78(15):1541–9. 10.1016/j.jacc.2021.08.017 34620412

[23] van ‘t Veer M Adjedj J Wijnbergen I Tóth GG Rutten MC Barbato E Novel monorail infusion catheter for volumetric coronary blood flow measurement in humans: in vitro validation. EuroIntervention. (2016) 12(6):701–7. 10.4244/EIJV12I6A114 27542781

[24] Kobayashi Y Fearon WF Honda Y Tanaka S Pargaonkar V Fitzgerald PJ Effect of sex differences on invasive measures of coronary microvascular dysfunction in patients with angina in the absence of obstructive coronary artery disease. JACC: Cardiovascular Interventions. (2015) 8(11):1433–41. 10.1016/j.jcin.2015.03.045 26404195 PMC5292322

[25] Chung J-H Lee Kyung E Lee Joo M Her A-Y Kim Chee H Choi Ki H Effect of sex difference of coronary microvascular dysfunction on long-term outcomes in deferred lesions. JACC Cardiovasc Interv. (2020) 13(14):1669–79. 10.1016/j.jcin.2020.04.002 32593698

[26] Nardone M McCarthy M Ardern CI Nield LE Toleva O Cantor WJ Concurrently low coronary flow reserve and low Index of microvascular resistance are associated with elevated resting coronary flow in patients with chest pain and nonobstructive coronary arteries. Circ Cardiovasc Interv. (2022) 15(3):e011323. 10.1161/CIRCINTERVENTIONS.121.011323 35135301

[27] Fournier S Keulards DCJ van ‘t Veer M Colaiori I Di Gioia G Zimmermann FM Normal values of thermodilution-derived absolute coronary blood flow and microvascular resistance in humans. EuroIntervention. (2020) 17(4):e309–e16. 10.4244/EIJ-D-20-00684 PMC9724861 33016881

[28] Demir OM Boerhout CKM de Waard GA van de Hoef TP Patel N Beijk MAM Comparison of Doppler flow velocity and thermodilution derived indexes of coronary physiology. JACC Cardiovasc Interv. (2022) 15(10):1060–70. 10.1016/j.jcin.2022.03.015 35589236 PMC9126183

[29] Gilligan DM Quyyumi AA Cannon R III. Effects of physiological levels of estrogen on coronary vasomotor function in postmenopausal women. Circulation. (1994) 89(6):2545–51. 10.1161/01.CIR.89.6.2545 8205663

[30] Cannon RO III Epstein SE. “microvascular angina” as a cause of chest pain with angiographically normal coronary arteries. Am J Cardiol. (1988) 61(15):1338–43. 10.1016/0002-9149(88)91180-0 3287885

[31] Knuuti J Wijns W Saraste A Capodanno D Barbato E Funck-Brentano C 2019 ESC guidelines for the diagnosis and management of chronic coronary syndromes: the task force for the diagnosis and management of chronic coronary syndromes of the European society of cardiology (ESC). Eur Heart J. (2019) 41(3):407–77. 10.1093/eurheartj/ehz425 31504439

[32] Gulati M Levy PD Mukherjee D Amsterdam E Bhatt DL Birtcher KK 2021 AHA/ACC/ASE/CHEST/SAEM/SCCT/SCMR guideline for the evaluation and diagnosis of chest pain: a report of the American college of cardiology/American heart association joint committee on clinical practice guidelines. Circulation. (2021) 144(22):e368–454. 10.1161/CIR.0000000000001029 34709879

[33] Doucette JW Corl PD Payne HM Flynn AE Goto M Nassi M Validation of a Doppler guide wire for intravascular measurement of coronary artery flow velocity. Circulation. (1992) 85(5):1899–911. 10.1161/01.CIR.85.5.1899 1572046

[34] Pijls NHJ De Bruyne B Smith L Aarnoudse W Barbato E Bartunek J Coronary thermodilution to assess flow reserve. Circulation. (2002) 105(21):2482–6. 10.1161/01.CIR.0000017199.09457.3D 12034653

[35] Gallinoro E Bertolone Dario T Fernandez-Peregrina E Paolisso P Bermpeis K Esposito G Reproducibility of bolus versus continuous thermodilution for assessment of coronary microvascular function in patients with ANOCA. EuroIntervention. (2023) 19(2):e155–e66. 10.4244/EIJ-D-22-00772 36809253 PMC10242662

[36] Taylor DJ Feher J Halliday I Hose DR Gosling R Aubiniere-Robb L Refining our understanding of the flow through coronary artery branches; revisiting Murray’s law in human epicardial coronary arteries. Front Physiol. (2022) 13:871912. 10.3389/fphys.2022.871912 35600296 PMC9119389

[37] Sturdy J Kjernlie JK Nydal HM Eck VG Hellevik LR. Uncertainty quantification of computational coronary stenosis assessment and model based mitigation of image resolution limitations. J Comput Sci. (2019) 31:137–50. 10.1016/j.jocs.2019.01.004

[38] Tar B Ágoston A Üveges Á Szabó GT Szűk T Komócsi A Pressure- and 3D-derived coronary flow reserve with hydrostatic pressure correction: comparison with intracoronary Doppler measurements. J Pers Med. (2022) 12(5):780. 10.3390/jpm12050780 35629202 PMC9146986

[39] Üveges Á Tar B Jenei C Czuriga D Papp Z Csanádi Z The impact of hydrostatic pressure on the result of physiological measurements in various coronary segments. Int J Cardiovasc Imaging. (2021) 37(1):5–14. 10.1007/s10554-020-01971-w 32804319 PMC7878210

