# Supplementary material for: Sex differences in coronary microvascular resistance measured by a computational fluid dynamics model
Source: Front Cardiovasc Med. 2023 Jul 6;10:1159160. doi: 10.3389/fcvm.2023.1159160 (PMC10357508; doi:10.3389/fcvm.2023.1159160)
Supplement: Supplementary file 1 [file Table1.docx]

Supplementary material

# *
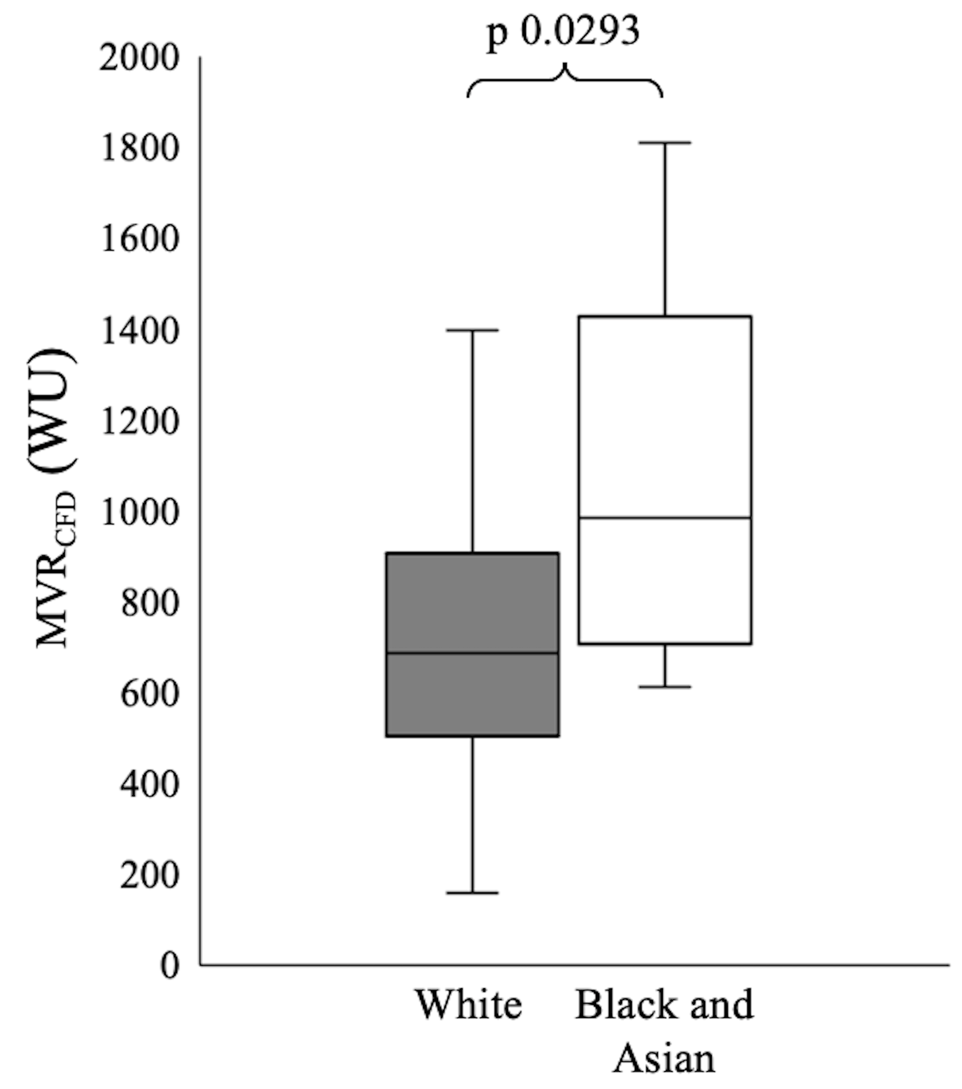
*

# *Supplementary figure 1.* C*MVR_CFD_ was significantly higher in black and Asian patients versus white Caucasian patients*
